# COVID-19 Treatments Sold Online Without Prescription Requirements in the United States: Cross-sectional Study Evaluating Availability, Safety and Marketing of Medications

**DOI:** 10.2196/27704

**Published:** 2022-02-16

**Authors:** Sachiko Ozawa, Joanna Billings, Yujiao Sun, Sushan Yu, Benjamin Penley

**Affiliations:** 1 Division of Practice Advancement and Clinical Education Eshelman School of Pharmacy University of North Carolina at Chapel Hill Chapel Hill, NC United States; 2 Department of Maternal and Child Health Gillings School of Global Public Health University of North Carolina at Chapel Hill Chapel Hill, NC United States

**Keywords:** COVID-19, medication, internet, online pharmacy, drug

## Abstract

**Background:**

The COVID-19 pandemic has increased online purchases and heightened interest in existing treatments. Dexamethasone, hydroxychloroquine, and lopinavir-ritonavir have been touted as potential COVID-19 treatments.

**Objective:**

This study assessed the availability of 3 potential COVID-19 treatments online and evaluated the safety and marketing characteristics of websites selling these products during the pandemic.

**Methods:**

A cross-sectional study was conducted in the months of June 2020 to August 2020, by searching the first 100 results on Google, Bing, and Yahoo! mimicking a US consumer. Unique websites were included if they sold targeted medicines, were in English, offered US shipping, and were free to access. Identified online pharmacies were categorized as rogue, unclassified, or legitimate based on LegitScript classifications. Patient safety characteristics, marketing techniques, price, legitimacy, IP addresses, and COVID-19 mentions were recorded.

**Results:**

We found 117 websites: 30 selling dexamethasone (19/30, 63% rogue), 39 selling hydroxychloroquine (22/39, 56% rogue), and 48 selling lopinavir-ritonavir (33/48, 69% rogue). This included 89 unique online pharmacies: 70% were rogue (n=62), 22% were unapproved (n=20), and 8% were considered legitimate (n=7). Prescriptions were not required among 100% (19/19), 61% (20/33), and 50% (11/22) of rogue websites selling dexamethasone, lopinavir-ritonavir, and hydroxychloroquine, respectively. Overall, only 32% (24/74) of rogue websites required prescriptions to buy these medications compared with 94% (31/33) of unapproved and 100% (10/10) of legitimate websites (*P*<.001). Rogue sites rarely offered pharmacist counseling (1/33, 3% for lopinavir-ritonavir to 2/22, 9% for hydroxychloroquine). Drug warnings were unavailable in 86% (6/7) of unapproved dexamethasone sites. It was difficult to distinguish between rogue, unapproved, and legitimate online pharmacies solely based on website marketing characteristics. Illegitimate pharmacies were more likely to offer bulk discounts and claim price discounts, yet dexamethasone and hydroxychloroquine were more expensive online. An inexpensive generic version of lopinavir-ritonavir that is not authorized for use in the United States was available online offering US shipping. Some websites claimed hydroxychloroquine and lopinavir-ritonavir were effective COVID-19 treatments despite lack of scientific evidence. In comparing IP addresses to locations claimed on the websites, only 8.5% (7/82) matched their claimed locations.

**Conclusions:**

The lack of safety measures by illegitimate online pharmacies endanger patients, facilitating access to medications without appropriate oversight by health care providers to monitor clinical response, drug interactions, and adverse effects. We demonstrated how easy it is to go online to buy medications that are touted to treat COVID-19 even when current clinical evidence does not support their use for self-treatment. We documented that illegitimate online pharmacies sidestep prescription requirements, skirt pharmacist counseling, and make false claims regarding efficacy for COVID-19 treatment. Health care professionals must urgently educate the public of the dangers of purchasing drugs from illegitimate websites and highlight the importance of seeking treatment through authentic avenues of care.

## Introduction

The coronavirus disease 2019 (COVID-19) pandemic has intensified the demand for effective medications and made online purchases commonplace. According to a recent global study of 5000 consumers, over one-third (36%) of consumers reported shopping online weekly since the pandemic, which is up from 28% pre-COVID-19 [1]. As online shopping becomes more common, the rate of online purchase of medications is also increasing [2]. Online pharmacies have grown from US $29.35 billion in 2014 to a projected global market size of US $128 billion by 2023 [3].

Yet, consumers may not be aware of how prevalent illicit pharmacies are online. In 2016, there were 30,000-35,000 online pharmacies accessible to US customers on the internet [4]. Of these pharmacies, 96% were found to be illegitimate and in violation of US pharmacy laws and practice standards [5]. Illegitimate online pharmacies pose a significant safety threat to consumers, as their products cannot be guaranteed; the manufacturing, storage, and shipping conditions of the drugs may not be regulated; and they bypass pharmacists who can ensure proper dosages and use of medications [6].

As the pandemic continues to claim many lives [7], there has been heightened interest in finding potential treatments for COVID-19 among existing medications. Several existing medications have been touted as potential treatments for COVID-19, including dexamethasone, hydroxychloroquine, and lopinavir-ritonavir [8]. Dexamethasone is a glucocorticoid agent used for several disease states for its anti-inflammatory and immunosuppressive properties. It has been trialed in patients with COVID-19 to see if it may reduce disease progression to respiratory failure and death [9]. A controlled, open-label trial among patients who were hospitalized with COVID-19 found that the use of dexamethasone resulted in lower 28-day mortality among those who were receiving either invasive mechanical ventilation or oxygen alone [9,10]. This trial, along with others, supports the idea that dexamethasone can prevent mortality in hospitalized patients with COVID-19 [11]. Such supportive evidence behind the effectiveness of dexamethasone has caused an increase in its use [12]. It is important to note though that corticosteroids such as dexamethasone are not benign drugs and should only be taken under the direct supervision of a medical provider. If misused, dexamethasone can cause side effects such as hypertension, endocrine abnormalities, vision problems, pancreatitis, and osteoporosis [13]. Monitoring the use of dexamethasone is therefore required to assess side effects and response to therapy.

Hydroxychloroquine has also been considered a potential COVID-19 treatment and prophylaxis [14]. Hydroxychloroquine is an aminoquinoline anti-infective agent indicated for the treatment of malaria. Despite promising mechanistic properties, several randomized clinical trials found that hydroxychloroquine did not prevent illness or confirmed infection when used as postexposure prophylaxis within 4 days after moderate or high-risk exposure to COVID-19 [14,15]. However, endorsement of the medication by high-profile figures including former US President Donald Trump during early phases of the pandemic drew public attention to the medication. It was reported that Google search volume for purchasing hydroxychloroquine increased by 14-fold in March 2020, when the clinical efficacy for the medication was still inconclusive [16]. The use of hydroxychloroquine for COVID-19 can be problematic especially without a health care prescription, putting patients at risk for insufficient laboratory monitoring and side effects such as nausea, low blood sugar, movement disorders, eye problems, and cardiac problems [10].

Another medication that has been touted for COVID-19 treatment is lopinavir-ritonavir. Lopinavir-ritonavir is an antiretroviral agent that is used for HIV infection suppression. Since lopinavir had in vitro inhibitory activity against the SARS-CoV virus in 2003 and the SARS-CoV-2 (COVID-19) virus is genetically similar, clinical trials were conducted in patients with COVID-19 [17]. However, a randomized, controlled, open-label trial involving hospitalized adult patients with severe COVID-19 found no benefit of lopinavir-ritonavir treatment beyond standard care [14]. Further studies and case reports have reinforced these findings, and lopinavir-ritonavir is generally not recommended for treatment of COVID-19 [18,19]. If consumers use lopinavir-ritonavir in efforts to combat a COVID-19 infection, they would not only be using an ineffective treatment but would also be exposing themselves to unnecessary side effects.

We sought to assess the accessibility of dexamethasone, hydroxychloroquine, and lopinavir-ritonavir online during the COVID-19 pandemic and to analyze their implications for medication safety. In doing so, we recorded medication prices, marketing practices, and statements related to COVID-19 from websites selling these medications. Our aim was to assess what US consumers would find if they utilized online pharmacies looking for medications to treat COVID-19 amid the pressures of a global pandemic.

## Methods

We searched for online pharmacy websites selling dexamethasone, hydroxychloroquine, or lopinavir-ritonavir using 3 search engines (Google, Bing, and Yahoo!) between June 2020 and August 2020. Two searches were conducted on each search engine using the brand and generic names of each medication. We used the search terms “buy [drug name] online” (eg, “buy lopinavir-ritonavir online” and “buy Kaletra online”) to simulate patients seeking to buy these treatments online. We screened the first 100 search results for every search term and recorded the results of our searches. Websites were included in this analysis if they were published in the English language, were free to access, were active, claimed to sell the drugs of interest, had a unique URL, and were classified by LegitScript. Websites were excluded if they did not sell the medications of interest or did not ship to the United States. Screenshots of the web pages were taken at the time the pages were accessed.

The legitimacy of included websites was assessed by LegitScript, an online service that monitors online pharmacies for compliance with applicable laws and regulations. LegitScript classifies online pharmacies based on licensure or registration in appropriate jurisdictions, sales of controlled substances, history of disciplinary sanctions, requirement of valid prescription, compliance with applicable laws, protection of privacy, patient services offered, transparency, and domain name registration [4]. This analysis utilized LegitScript’s classification, where *rogue* pharmacies “engage in illegal, unsafe, or misleading activities like selling prescription drugs without a prescription,” *unapproved* pharmacies face “some problem with regulatory compliance or risk, such as operating legally in one jurisdiction but not in others,” and *legitimate pharmacies* “passed LegitScript’s certification criteria” [4]. Rogue and unapproved pharmacies were considered illegitimate pharmacies in this analysis.

In order to understand how online pharmacies attend to patient safety, we assessed whether prescriptions were required to purchase these medications or if patients needed to fill out a health-related questionnaire prior to ordering medications. We assessed whether pharmacist counseling was available, appropriate drug information was on the drug product page, warnings in using the medications were noted, and there was a quantity limit on purchases. Quantity control was defined as the website setting a maximum allowable quantity (or day supply) of medication that was available for purchase on the checkout page. Claims made related to the efficacy of these medications in treating COVID-19 were also abstracted from the websites. Patient safety analyses were conducted for each drug individually. Chi-squared tests were conducted to examine differences in patient safety characteristics by pharmacy legitimacy and across medications.

We recorded some marketing characteristics of websites, including whether the website claimed a price discount compared with brick-and-mortar pharmacies, availability of bulk product price discounts, and offers of coupons or promotion codes. Marketing characteristics of rogue, unapproved, and legitimate sites were compared using chi-squared tests. We tracked whether online pharmacies offered phone numbers or WhatsApp accounts and whether consumers could speak with sales associates. Websites that claimed to be registered with pharmacy-governing organizations were recorded, as well as customer testimonies and privacy assurances. Marketing of COVID-19–related products such as ads for masks, sanitizing wipes, and hand sanitizers, as well as explicit mentions of COVID-19 on websites, were also recorded. Quotes about COVID-19 from these websites were classified by website legitimacy and were analyzed qualitatively, examining themes that emerged from texts. Unique websites were defined as those with unique URLs, and marketing analyses were conducted once per unique website (ie, if a website sold 2 of our medications of interest, we reported the website’s marketing characteristic once).

Prices and shipping costs for obtaining 60 dexamethasone 0.5 mg tablets, hydroxychloroquine 200 mg capsules, and lopinavir-ritonavir 200 mg-50 mg tablets were collected from each website. These quantity and strengths were chosen as they were the most commonly available for patients to select and purchase presented on the web pages. Online pharmacy prices at checkout were compared with prices offered through GoodRx, which tracks prescription drug prices in the United States. Where possible, we compared prices of generic versions of the medications. However, the GoodRx price for lopinavir-ritonavir reflected the price for the brand name drug Kaletra in the United States, as a generic version had not been approved for use in the United States. Country locations reported on pharmacy websites were also recorded and compared with registered locations of the IP addresses identified via IP2location [20]. Where available, we assessed the monthly traffic volume of pharmacy sites using SimilarWeb, a site that specializes in web analytics and reports traffic volumes from open exchanges of first-party data, and by surveying public data sources [21].

## Results

Across 3 search engines, we identified a total of 117 websites that claimed to sell dexamethasone, hydroxychloroquine, or lopinavir-ritonavir online. This included 30 websites selling dexamethasone, 39 sites selling hydroxychloroquine, and 48 sites selling lopinavir-ritonavir. The majority of websites were illegitimate—we only found 4 (4/30, 13%), 5 (5/39, 13%), and 1 (1/48, 2%) legitimate websites selling dexamethasone, hydroxychloroquine, and lopinavir-ritonavir, respectively. We found that 63% (19/30) of websites selling dexamethasone, 56% (22/39) of sites selling hydroxychloroquine, and 69% (33/48) of sites selling lopinavir-ritonavir were rogue based on LegitScript classifications. An additional 26 websites that were not classified by LegitScript were excluded from our analysis.

Rogue websites tended to be distinct from unapproved (31/33, 94%) and legitimate sites (10/10, 100%), with only 32% (24/74) of sites requiring prescriptions to buy these medications (*P*<.001; Table 1). Health-related questionnaires were seldom used across all websites (utilized by 1 rogue and 7 unapproved websites). Overall, few (15/117, 12.8%) websites made overt offers for consumers to speak with a pharmacist, including legitimate sites (1/10, 10%). Medication warnings were often missing from unapproved websites (13/33, 39%; *P*<.001). Quantity control was rarely seen in legitimate sites, as prescriptions were meant to limit purchasable quantity.

**Table 1:** Patient safety characteristics stratified by rogue, unapproved, and legitimate online pharmacies.

Patient safety characteristics	Rogue (n=74), n (%)	Unapproved (n=33), n (%)	Legitimate (n=10), n (%)	*P* value
Prescription required	24 (32)	31 (94)	10 (100)	<.001
Health-related questionnaire	1 (1)	7 (21)	0 (0)	<.001
Offer to speak with pharmacist	4 (5)	10 (30)	1 (10)	.002
Drug information on product page	44 (59)	18 (55)	9 (90)	.12
Drug related warnings on product page	60 (81)	13 (39)	8 (80)	<.001
Quantity control	45 (61)	27 (82)	2 (20)	.001

Across medications, many illegitimate websites did not require prescriptions to obtain dexamethasone (21/26, 81%), compared with hydroxychloroquine (11/34, 32%) and lopinavir-ritonavir (20/47, 43%; *P*=.02; Table 2). Rogue websites by far had the most lenient prescription requirements, with none (0/19, 0%) of the rogue websites selling dexamethasone requiring prescriptions, while 50% (11/22) and 39% (13/33) of rogue websites selling hydroxychloroquine and lopinavir/ritonavir, respectively, required prescriptions. Medication information and warnings were particularly lacking for illegitimate websites selling hydroxychloroquine and lopinavir-ritonavir, with only 65% (22/34) and 36% (17/47) of websites, respectively, providing accurate drug information. Among illegitimate websites, 53% (18/34) selling hydroxychloroquine and 77% (36/47) selling lopinavir-ritonavir provided medication-related warnings. Across all websites selling dexamethasone, 87% (26/30) included medicine information, and 73% (22/30) provided medication-related warnings on their web pages.

**Table 2:** Patient safety characteristics stratified by online pharmacy legitimacy and medication.

Patient safety characteristics	Rogue, n (%)				Unapproved, n (%)				Legitimate, n (%)				*P* value
	DEX^a^ (n=19)	HCQ^b^ (n=22)	L-R^c^ (n=33)	DEX (n=7)		HCQ (n=12)	L-R (n=14)	DEX (n=4)		HCQ (n=5)	L-R (n=1)		
Prescription required(yes)	0 (0)	11 (50)	13 (39)	5 (71)		12 (100)	14 (100)	4 (100)		5 (100)	1 (100)	.02	
Health-related questionnaire (yes)	0 (0)	1 (5)	0 (0)	0 (0)		3 (25)	4 (29)	0 (0)		0 (0)	0 (0)	.29	
Offer to speak with pharmacist(yes)	1 (5)	2 (9)	1 (3)	1 (14)		6 (50)	3 (21)	0 (0)		1 (20)	0 (0)	.87	
Drug information on product page(yes)	18 (95)	14 (64)	12 (36)	5 (71)		8 (67)	5 (36)	3 (75)		5 (100)	1 (100)	.59	
Drug-related warnings on product page (yes)	18 (95)	12 (54)	30 (91)	1 (14)		6 (50)	6 (43)	3 (75)		4 (80)	1 (100)	.06	
Quantity control (yes)	15 (79)	11 (50)	19 (58)	7 (100)		12 (100)	8 (57)	1 (25)		1 (20)	0 (0)	.37	

^a^DEX: dexamethasone.

^b^HCQ: hydroxychloroquine.

^c^L-R: lopinavir-ritonavir.

Marketing characteristics were examined across unique websites. We identified 89 unique websites that were classified by LegitScript, as 28 websites sold more than one product of interest. Among the 89 websites, 62 (70%) were identified as rogue, 20 (22%) were unapproved, and only 7 (8%) websites were legitimate.

When examining marketing characteristics of websites, it was more difficult to distinguish between rogue, unapproved, and legitimate online pharmacies (Table 3). Privacy assurances were most frequently offered by unapproved pharmacies (17/20, 85%), compared with 52% (32/62) for rogue and 57% (4/7) for legitimate pharmacies (*P*=.03). Rogue and unapproved websites appeared to be more likely to claim price discounts (58/62, 94% and 18/20, 90% respectively), while 71% (5/7) of legitimate websites made a similar claim (*P*=.12). Rogue and unapproved websites tended to offer more bulk discounts (41/62, 66% and 13/20, 65%, respectively) compared with legitimate websites (2/7, 29%; *P*=.15). It is notable that 32% (20/62) of rogue websites and 60% (12/20) of unapproved websites claimed to be registered by pharmacy-governing organizations. The most common registration claims made by illegitimate websites included the Canadian International Pharmacy Association (CIPARx; 33/82, 40%) and PharmacyChecker (25/82, 30%). Legitimate websites mostly claimed registration with the National Association of Boards of Pharmacy (2/7, 29%), the Better Business Bureau (4/7, 57%), or LegitScript (5/7, 71%).

**Table 3:** Marketing characteristics stratified by rogue, unapproved, and legitimate online pharmacies (n=89).

Marketing characteristics	Rogue (n=62), n (%)	Unapproved (n=20), n (%)	Legitimate (n=7), n (%)	*P* value^a^
Claims price discount (yes)	58 (94)	18 (90)	5 (71)	.12
Bulk discounts (yes)	41 (66)	13 (65)	2 (29)	.15
Coupon or promo code (yes)	37 (60)	8 (40)	4 (57)	.29
Phone # or WhatsApp (yes)	56 (90)	19 (95)	7 (100)	.56
Offer to speak with an associate (yes)	45 (72)	16 (80)	6 (86)	.60
Registration claims (yes)	20 (32)	12 (60)	3 (43)	.08
Customer testimonials (yes)	39 (63)	8 (40)	5 (71)	.15
Privacy claims (yes)	32 (52)	17 (85)	4 (57)	.03

^a^Analysis was conducted across websites selling dexamethasone, hydroxychloroquine, and/or lopinavir-ritonavir, which were classified by LegitScript for legitimacy.

A total of 7 illegitimate websites made unsubstantiated claims regarding the efficacy of hydroxychloroquine (n=4) or lopinavir-ritonavir (n=3) in treating COVID-19 (Table 4). Illegitimate pharmacies made a wide variety of uncorroborated medication efficacy claims from “hydroxychloroquine has shown positive results when being used to treat patients with COVID-19” to “[lopinavir-ritonavir] has been tested and included in the protocols of treatment of the novel coronavirus COVID-19.” One illegitimate website quoted former US President Donald Trump’s Twitter account to note that hydroxychloroquine should be used to treat COVID-19. Four illegitimate websites created a COVID-19 medication category and tagged hydroxychloroquine or lopinavir-ritonavir. Illegitimate websites also noted supply limitations: “[Hydroxychloroquine is] not available at this time due to COVID-19 shipping disruptions.” COVID-19–related supplies such as gloves, masks, or test kits were advertised by 4 illegitimate websites.

On the other hand, legitimate pharmacy websites rarely mentioned COVID-19 on their web pages, neither claiming nor denying the efficacy of these medications related to COVID-19. Two legitimate websites mentioned limitations in dispensing hydroxychloroquine due to COVID-19, limiting supplies to existing hydroxychloroquine patients or restricting hydroxychloroquine to only patients with a positive COVID-19 test. One legitimate website advertised hand sanitizers and masks alongside these medications.

**Figure 1:**
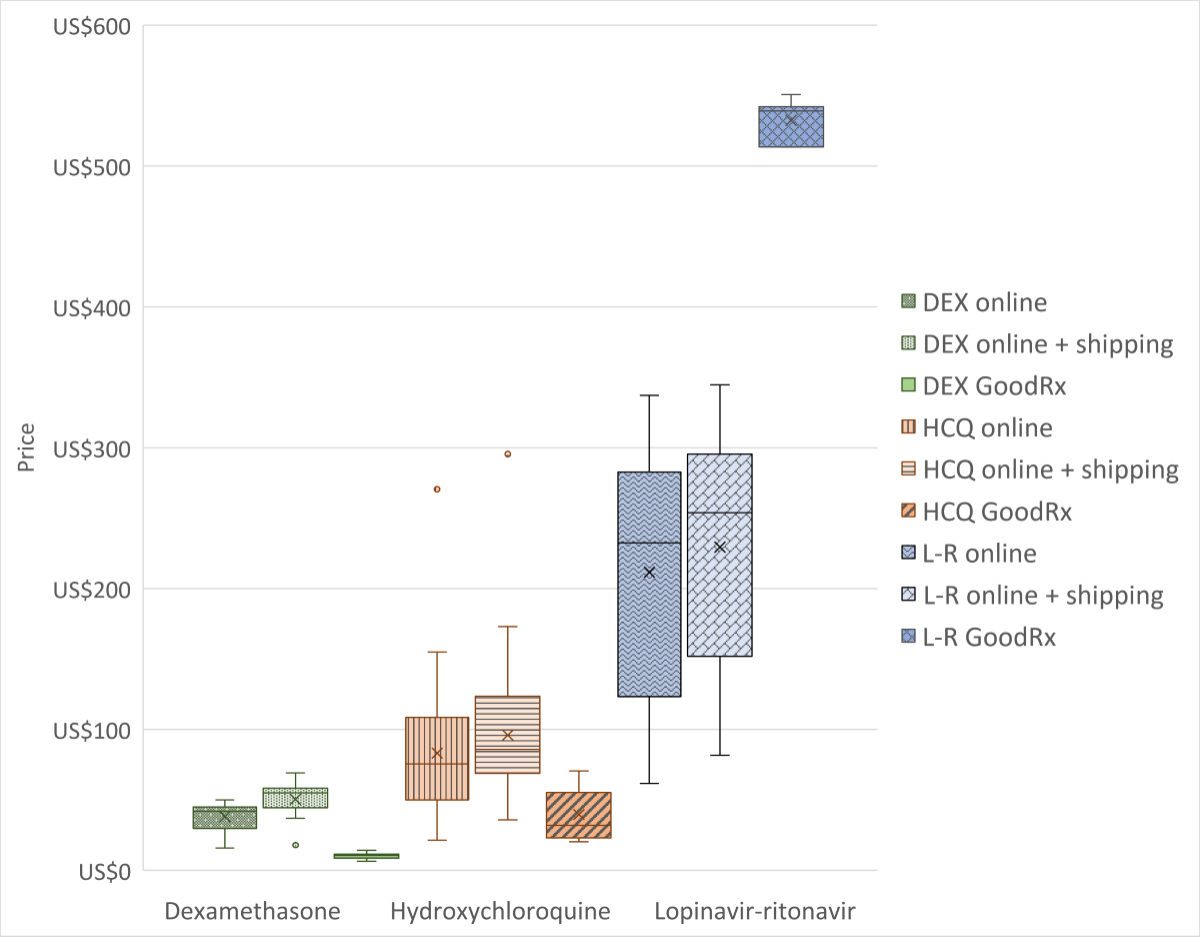
Prices to purchase 60 tablets/capsules of dexamethasone (DEX), hydroxychloroquine (HCQ), and lopinavir-ritonavir (L-R) online versus GoodRx.

Prices, with and without standard shipping, for 60 tablets of each medication bought online (0.5 mg tablets of dexamethasone, 200 mg capsules of hydroxychloroquine, and 200 mg lopinavir and 50 mg ritonavir tablets) were compared with the equivalent quantity price at brick-and-mortar pharmacies listed on GoodRx (Figure 1). Dexamethasone and hydroxychloroquine were both more expensive online, with or without shipping, compared with listed average GoodRx prices. Average prices were lower at brick-and-mortar stores for 0.5 mg tablets of dexamethasone at US $10.74, compared with US $38.07 for the medication online and US $50.54 with shipping. Similarly, 200 mg capsules of hydroxychloroquine were cheaper at brick-and-mortar stores at US $39.64 on average, compared with US $83.22 online and US $96.04 with shipping. A generic version of lopinavir-ritonavir was available online for US consumers and sold on average at US $211.77 (US $229.52 with shipping), even though this generic drug has not been approved for use in the United States. This unapproved generic lopinavir-ritonavir was much cheaper online than average GoodRx prices for brand-name drug Kaletra at US $532.40.

**Table 4:** COVID-19 mentions on illegitimate and legitimate online pharmacy websites.

Type of COVID-19 mentions		Quotes	Mentions^a^
**Quotes from illegitimate pharmacies**			
	Direct claims that medication may be effective for COVID-19	“drug [hydroxychloroquine] confirmed effective on COVID-19” “It was announced by American president that Hydroxychlorquine should be used to treat coronavirus, Donald Trump said this in his Twitter on March 21 2020.” “[study] proved that Hydroxychloroquine… can cut the death rate significantly in sick, hospitalized COVID patients without heart-related side effects... The analysis of the study shows hydroxychloroquine helped save lives.” “Hydroxychloroquinine has shown positive results when being used to treat patients with Covid-19 (coronavirus).” “it was found out that Kaletra shows positive results in a blockage of a COVID-19 viral replication” “This HIV medication [lopinavir-ritonavir] is one of the few drugs effective for COVID-19 treatment)” “It [lopinavir-ritanovir] has been tested and included in the protocols of treatment of the novel coronavirus COVID-19”	7
	Claims medication may be effective for COVID-19	“Reports have shown that this medicine [hydroxychloroquine] might be effective against Coronavirus, but it has not been proven” “Reports have shown that this medicine [lopinavir-ritonavir] might be effective against Coronavirus, but it has not been proven.”	2
	Supply/shipping limitation due to COVID-19	“…New Zealand, in an effort to preserve adequate stocks of this medicine for their own people during the Covid-19 pandemic have halted all exports until further notice” “Limited supply. Currently being dispensed to existing Plaquenil patients only” “Not available at this time due to COVID-19 shipping disruptions.”	3
	Precaution regarding efficacy in COVID-19	“Taking this medication will not prevent you from passing HIV or COVID-19 to other people”	1
	Medication included in COVID-19 medication category	Tag^b^: COVID-19 [hydroxychloroquine] Tag^b^: coronavirus [lopinavir-ritanovir] [hydroxychloroquine] Tag^b^: covid19 treatment, covid19 medications uk [lopinavir-ritanovir]	4
	Ads for products related to COVID-19	Gloves COVID test kits Masks	4
**Quotes from legitimate pharmacies**			
	Indication limitation or dispense restriction due to COVID-19	“we are ONLY dispensing Hydroxychloroquine to patients with history of use for an autoimmune disorder … prior to 3/1/2020 or a positive diagnosis of COVID-19… Doctors prescribing outside of their scope of practice will be DENIED” “Due to the national shortage, we can only dispense this drug [hydroxychloroquine] if you have tested positive for coronavirus/COVID-19.”	2
	Ads for products related to COVID-19	Sanitizer and masks	1

^a^Denotes the number of online pharmacy websites per type of COVID-19 mention.

^b^These websites created COVID-19 medication categories or categorized the medication under a specific tag on the product pages.

**Figure 2:**
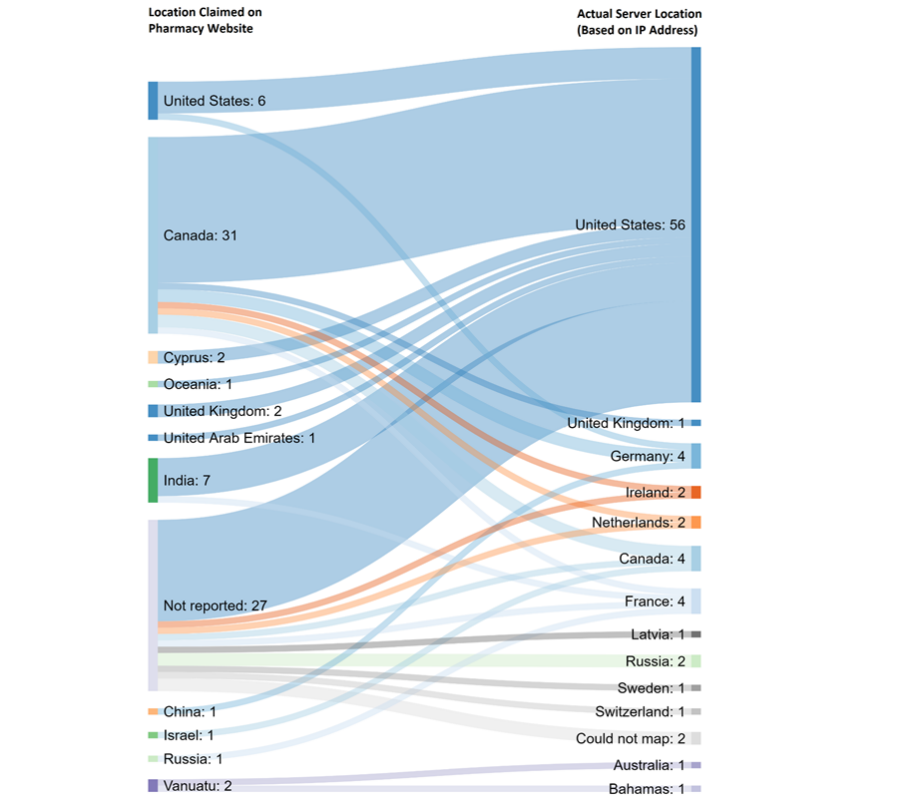
Reported versus server locations of illegitimate online pharmacies (n=82).

Large numbers of countries were involved in hosting online pharmacies, either where online pharmacies claimed to be or where their servers were located (Figure 2). Most of the illegitimate websites (75/82, 92%) hosted their servers in a country different from that listed on their website. The majority of illegitimate websites (58/82, 71%) either claimed to be a Canadian pharmacy (31/82, 38%) or did not list any country location (27/82, 33%) on their websites. Of the websites claiming to be located in Canada or India (38/82, 46%), the majority actually had IP addresses with a registered server in the United States (29/38, 76%). All legitimate websites (n=7) claimed to be in the United States and had IP addresses that indicated US servers. Website traffic data were available on SimilarWeb for 9 websites (3 rogue and 6 legitimate sites). Average website traffic for legitimate websites ranged from 10,200 to 10,900,000 monthly views, while rogue websites ranged from 10,000 to 20,000 monthly views.

## Discussion

### Principal Findings

We found that illegitimate pharmacy websites claiming to sell dexamethasone, hydroxychloroquine, and lopinavir-ritonavir endanger patient safety by bypassing prescription requirements, denying pharmacist counseling, and making false claims regarding efficacy in treating COVID-19 infections. We demonstrated that these websites are easy to find using common search engines and that they attempt to garner attention from potential consumers by claiming discounted prices and offering bulk discounts. We discuss in the following sections the reasons why these websites are harmful (lack of prescriptions, no pharmacist contact, and use of these medications for COVID-19 treatment) and how they try to lure consumers (medicine prices, online pharmacy marketing, and global transactions) [22].

### Lack of Prescriptions

Many illegitimate websites we found selling dexamethasone (21/26, 81%), hydroxychloroquine (11/34, 32%), and lopinavir-ritonavir (20/47, 43%) did not require prescriptions. This is a recipe for tragedy as prescriptions ensure that health care providers have reviewed patients’ medical histories and that selected medications are safe and appropriate. The lack of prescriptions needed to access these medications through online pharmacies is an immense patient safety concern, as these medications require intensive monitoring for side effects and therapeutic effects when they are initiated.

For example, dexamethasone requires a physician-guided dosing taper for safe and effective use. If dexamethasone is stopped abruptly after long-term use, it can cause hypothalamic-pituitary adrenal axis suppression, which can lead to a decreased immune response against infection or trauma [13]. Moreover, side effects of taking dexamethasone include hypertension, endocrine abnormalities, vision problems, pancreatitis, and osteoporosis [13]. For hydroxychloroquine, there have been reports of fatal cardiomyopathy and arrythmias with its use, which have led to recommendations for intensive cardiac monitoring during therapy initiation [23]. Hydroxychloroquine also has the potential to cause adverse effects to the liver, kidney, cerebellar cortex, pancreas, and eyes [23,24]. Lopinavir-ritonavir requires provider guidance for safe dosing and administration. An overdose of lopinavir-ritonavir can result in cardiac toxicity, renal failure, and central nervous system depression [25].

### No Pharmacist Contact

We also found that very few websites (15/117, 12.8%) offered pharmacist counseling before consumers purchase these medications, including even legitimate sites (1/10, 10%). Pharmacists ensure medication appropriateness and safety by ensuring that patients have the opportunity to clarify any dosing instructions, interactions, or side effects before they take medications [26]. With no pharmacist oversight, patients are more likely to experience drug interactions, incorrect dosing, or side effects that could have been avoided with counseling. For example, previous studies have examined the association between online pharmacy use, medication use, and health outcomes, where patients who obtained medications online were more likely to use medications at higher doses and more frequently and experience adverse events compared with patients obtaining medications from brick-and-mortar stores [27]. Additionally, most states in the United States have regulations in place where an offer to counsel with a pharmacist must be made at the time of medication dispensing [28]. Buying medications through online pharmacies bypasses these important patient safety measures and regulations.

Some adverse events specific to these medications could be avoided if pharmacists are able to review patients’ profiles to analyze for interacting medications and counsel patients on potential side effects. For example, patients taking dexamethasone should not take nonsteroidal anti-inflammatory drugs as using both increases the risk of gastrointestinal side effects [13]. Since dexamethasone is metabolized in the liver, other medications that affect hepatic enzymes can also affect the metabolism of dexamethasone [13]. Likewise, hydroxychloroquine should also be used with caution in patients taking hepatotoxic medications or with hepatic disease [23]. Since hydroxychloroquine and lopinavir-ritonavir can increase the risk for cardiac arrythmia, it is important to avoid other medications that have a similar cardiac effect where possible [23,25]. Moreover, lopinavir-ritonavir interacts with any medications that induce, inhibit, or are metabolized by CYP3A, a very common metabolic liver enzyme [25]. If taken with a medication that is primarily cleared by CYP3A, lopinavir-ritonavir can cause buildup of the medication, which can lead to life-threatening side effects [25]. These examples demonstrate the importance for all online pharmacies to facilitate consumer interactions with pharmacists.

### Use of These Medications for COVID-19 Treatment

Although some online pharmacy sites have touted the efficacy of these medications in treating COVID-19, current clinical evidence does not support their use for self-treatment of COVID-19 infections. Dexamethasone was found to be effective in reducing mortality in patients with COVID-19 in the RECOVERY trial; however, the benefit was only seen among patients with severe disease requiring supplemental oxygen or invasive mechanical ventilation [9]. Online purchasing of dexamethasone for self-treatment of COVID-19 is therefore not appropriate.

One of the most common references to medicine efficacy for COVID-19 treatment on pharmacy websites was related to hydroxychloroquine (n=14). Four illegitimate websites claimed hydroxychloroquine as an effective treatment for COVID-19, with 1 site citing former US President Trump’s Twitter account as the source for this claim rather than scientific sources. Other sites created a COVID-19 medication category and deceptively included hydroxychloroquine in the category page. Hydroxychloroquine was one of the most studied medications during the early phase of the pandemic, with more than 50 registered clinical trials by May 2020 [29,30]. On April 27, 2020, the Food and Drug Administration (FDA) issued an emergency use authorization (EUA) for the use of hydroxychloroquine to treat adults and adolescents hospitalized with COVID-19 who are not able to participate in clinical trials. However, this EUA was revoked on June 15, 2020 after the FDA reviewed clinical evidence [31]. The FDA concluded that hydroxychloroquine is unlikely to be effective and the potential benefit of using the treatment no longer outweighs the risks of serious cardiac adverse events and other side effects associated with the medication [31]. The RECOVERY trial in the United Kingdom found no mortality benefit with hydroxychloroquine, while the Solidarity Trial coordinated by the World Health Organization terminated the hydroxychloroquine arm early based on interim analysis results that showed little or no reduction of mortality in hospitalized patients with COVID-19 [18,32].

We found one illegitimate website falsely claiming that lopinavir-ritonavir showed positive results in blocking COVID-19 viral replication. Such false statements can be hazardous for patients seeking reliable information. Similar to hydroxychloroquine, lopinavir-ritonavir was a popular explorative treatment at first, as a systematic review in 2020 including 9152 hospitalized patients found that it was the most frequently administered treatment, received by 21.9% of patients [33]. However, the RECOVERY trial also found the medication to not be effective, and the Solidarity Trial discontinued its lopinavir-ritonavir arm based on an interim analysis showing similar results [18,19].

### Medicine Prices

Prior studies have suggested that the cost of medications online vis-à-vis brick-and-mortar stores differs depending on the product [34-37]. Here, we report similar findings. While the majority (76/82, 93%) of illegitimate online pharmacies claimed price discounts on their products, it was cheaper on average to purchase dexamethasone and hydroxychloroquine at brick-and-mortar pharmacies. We found that average online prices (excluding shipping) for dexamethasone and hydroxychloroquine were 285% and 110% more expensive, respectively, than GoodRx.

On the other hand, prices for lopinavir-ritonavir were cheaper on online pharmacies. In the United States, lopinavir-ritonavir 200 mg-50 mg tablets are only available as a brand name product, Kaletra, at an average GoodRx price of US $532.40. Meanwhile, illegitimate online pharmacies advertised a generic lopinavir-ritonavir that has not been approved in the United States at an average price of US $211.77. The availability of an unapproved and cheaper “generic” lopinavir-ritonavir through online pharmacies means US consumers could in fact obtain a different medicine, a generic drug not approved in the United States, with significant safety concerns.

### Online Pharmacy Marketing

Illegitimate websites were more likely to claim price discounts (76/82, 93%) than legitimate websites (5/7, 71%). They were also more likely to offer bulk discounts if the customer placed larger orders. Many illegitimate websites also made claims to be registered by pharmacy-governing organizations, claiming to be approved by various sites as a legitimate business practice. These registration claims often cited CIPARx or PharmacyChecker. It is important to note that LegitScript has found that CIPARx and PharmacyChecker have previously given approval to websites legally found to be illegitimate [17]. The lack of reliability of such accreditation sites are problematic and need to be corrected as less savvy customers may see a symbol of accreditation and believe the website to be legitimate.

### Global Transactions

Most of the illegitimate online pharmacy websites analyzed in this study (75/82, 92%) hosted their servers in a country different from that listed on their website. This is in line with other studies that have reported similar differences in server and listed locations [35]. Although this difference does not verify that online pharmacies’ physical locations are different from what are reported, it does raise the concern that many of these pharmacies are misleading patients by reporting to be located in one country but are actually dispensing from another country. The discrepancies also increase barriers to enforcement agencies’ regulations. In addition, the pharmacies may be obtaining their medications from international manufacturers and suppliers that do not comply with US FDA regulations and good manufacturing practices. Worse yet, the medications dispensed by these illegitimate online pharmacies may be substandard or falsified and could cause harm to patients [38]. As 1 in 10 essential medications sold in low- and middle-income countries have been found to be substandard or falsified, some medications sold through online pharmacies could be substandard or falsified [39,40]. Global transactions facilitated by online pharmacies can be an especially opportunistic market during pandemics, when demand for COVID-19 treatments is high and regulatory actions may be inadequate [41]. Interventions are needed globally to ensure access to safe, quality-assured, and effective medications during the COVID-19 pandemic and beyond, with protections to minimize medication harm.

There is a number of limitations of our study to note. First, we did not attempt to capture the entirety of the online pharmacy landscape and were only able to present a snapshot of results at a point in time. We utilized 3 most commonly used search engines, conducted searches from the United States, conducted searches from the United States, and included unique sites from the first 10 pages of search results, imitating the behavior of a typical US consumer who may try to find online pharmacy websites selling these medications. We present a cross-sectional study where our data collection occurred in the summer of 2020. There may have been temporal changes to the availability of these medications and alterations in marketing tactics employed by online pharmacies since then. Second, this study focused solely on dexamethasone, hydroxychloroquine, and lopinavir-ritonavir, when the demand and supply for other medications to treat COVID-19 may have also been affected during this pandemic. Our medications of study were chosen based on their high publicity as a potential COVID-19 treatment during the first half of 2020.

We were also unable to uncover the actual purchasing frequency on these sites. We note that the web traffic to illegitimate online pharmacies may not be reflective of actual transaction volume, as many sites regularly close and open with new URLs or redirect links to avoid detection and regulation. However, the results we found demonstrate a snapshot of what an average US consumer may view while purchasing. Moreover, due to ethical concerns regarding providing fake prescriptions to these websites, we did not actually purchase medications from these online pharmacies. Therefore, we were unable to investigate the actual dispensing locations or test the quality of medications. Without following through with the full purchasing process, we could not confirm if pharmacist counseling is indeed offered after checkout. However, despite these limitations, we followed the systemic approach outlined in the methodology to assess the availability of high-profile medications during the COVID-19 pandemic from online pharmacies.

### Conclusion

This analysis illustrates how easy it is to go online to buy medications that are touted to treat COVID-19 even when current clinical evidence does not support their use for self-treatment. Illegitimate online pharmacies endanger patient safety by sidestepping prescription requirements, skirting pharmacist counseling, and making false claims regarding efficacy in treating COVID-19 infections. It is important for health care providers to recognize the ease of access to these medications and raise awareness about current clinical evidence especially during the pandemic. Health care providers should also educate the public about the risks of purchasing medications from illegitimate websites, highlighting the importance of prescriptions and appropriate monitoring of therapeutic response, drug interactions, and adverse effects by pharmacists [42]. The amount of illegitimate online pharmacies that surfaced with our search also suggests a need for better search algorithms and stricter monitoring and regulations to prevent illegitimate pharmacies from being easily accessible online.

## Abbreviations

CIPARx: Canadian International Pharmacy Association
EUA: Emergency Use Authorization
FDA: Food and Drug Administration
